# From Cortical Blindness to Conscious Visual Perception: Theories on Neuronal Networks and Visual Training Strategies

**DOI:** 10.3389/fnsys.2017.00064

**Published:** 2017-08-29

**Authors:** Vanessa Hadid, Franco Lepore

**Affiliations:** ^1^Département de Sciences Biomédicales, Université de Montréal Montréal, QC, Canada; ^2^Centre de Recherche en Neuropsychologie et Cognition (CERNEC), Département de Psychologie, Université de Montréal Montréal, QC, Canada

**Keywords:** cortical blindness, hemianopia, blindsight, neuronal substrates, global workspace theory, rehabilitation, multisensory training, visual training strategies

## Abstract

Homonymous hemianopia (HH) is the most common cortical visual impairment leading to blindness in the contralateral hemifield. It is associated with many inconveniences and daily restrictions such as exploration and visual orientation difficulties. However, patients with HH can preserve the remarkable ability to unconsciously perceive visual stimuli presented in their blindfield, a phenomenon known as blindsight. Unfortunately, the nature of this captivating residual ability is still misunderstood and the rehabilitation strategies in terms of visual training have been insufficiently exploited. This article discusses type I and type II blindsight in a neuronal framework of altered global workspace, resulting from inefficient perception, attention and conscious networks. To enhance synchronization and create global availability for residual abilities to reach visual consciousness, rehabilitation tools need to stimulate subcortical extrastriate pathways through V5/MT. Multisensory bottom-up compensation combined with top-down restitution training could target pre-existing and new neuronal mechanisms to recreate a framework for potential functionality.

## From Cortical Blindness to Awareness: Understanding Blindsight Beyond The First Sight

### Cortical Blindness

Normal vision in humans is primarily mediated by the geniculo-striate pathway where the visual information is processed in a hierarchical order via the retina, the lateral geniculate nucleus (LGN) and the striate cortex. Once the primary characteristics of visual information are processed, visual connections are sent to the parietal cortex involved in spatial attention and action (dorsal pathway) and to the temporal cortex involved in recognition and identification (ventral pathway). Following post-chiasmatic lesions inducing an alteration in the geniculo-striate pathway, a contralateral cortical blindness (CB) occurs, either as a result of a neurophysiologic disorder requiring surgical interventions of V1, or mainly subsequent to a stroke affecting the posterior visual cortex (Sand et al., 2013, for review, see Goodwin, 2014). Depending on the extent of the lesioned cortex, the visual field deficit may correspond to a CB of a few degrees (scotoma), a quarter of a hemifield (quadranopsia) or an entire hemifield (hemianopsia; for review see Swienton and Thomas, 2014). The homonymous hemianopsia (HH) is the most common visual cortical deficit representing 10% of stroke cases, with little more than 70,000 new cases per year in the US (Zhang et al., 2006; Mozaffarian et al., 2016). Moreover, more than 500,000 Americans live with a HH and this deficit reduces significantly their quality of life, for example preventing them from driving and, decreasing their reading, orientation and exploration visuo-spatial abilities (Perez and Chokron, 2014). In addition, due to comorbidity, HH significantly reduces the prognosis and the possibility of recovery from other damaged functions after stroke, including motor skills (Patel et al., 2000). As only a small minority experiences spontaneous recovery, possible within the first 6 months (Duquette and Baril, 2009), it is crucial to rehabilitate these individuals during this time. Unfortunately, with the exception of the vision restitution therapy (VRT) approved by the FDA due to its therapeutic potential (Sabel et al., 2005; Kasten et al., 2006), yet controversial on the benefits in terms of pure visual restitution (Horton, 2005a,b; Bouwmeester et al., 2007; Melnick et al., 2016), there are very few available resources for clinical interventions. This may be due to a lack of consensus in the literature caused by inconsistent results across the target population, differences between protocols used in research and an inefficient vision recovery (Pollock et al., 2011; for reviews see Riggs et al., 2007; Pouget et al., 2012). Furthermore, there is so much inter-individual variability in the origin and extent of lesions that most probably plasticity in the visual system is heterogeneous throughout the population. This leads us to the question: is visual recovery in cortically blind individuals possible with strategic rehabilitation? In theory, it would be conceivable, in the light of a well-documented preserved visual ability in CB referred to as blindsight, where visual information is processed in the blindfield without the knowledge of visual awareness (Weiskrantz et al., 1974).

In a behavioral perspective, blindsight is the dissociation between what is reported subjectively, for example, the subject states not seeing anything in the usual scale of binary report (seen, not seen), and what is measured objectively, i.e., the rate of correct answers in the two-alternative forced-choice paradigm is above the chance level. The first extended precepts of the phenomenon are described from the results obtained on GY who had a trauma affecting the striate cortex at a young age (Barbur et al., 1980; de Gelder et al., 1999; Kentridge et al., 2004) and DB who required a removal of V1 at an adult age (Weiskrantz, 1987; Tamietto et al., 2009). Blindsight includes the unconscious ability to be able to locate random targets by reaching or pointing at them, to determine the presence or absence of visual targets, to have a considerable visual acuity mainly for low spatial frequencies, to discriminate directions (Weiskrantz, 1986), to recognize colors (Brent et al., 1994), to detect global movement, to distinguish between coherence (Alexander and Cowey, 2009; Pavan et al., 2011) and to recognize facial expressions (de Gelder et al., 1999). However, it has been found that high contrast and fast movement stimuli could induce sensations described as something elusive that happened in the blind hemifield. Accordingly, a distinction has been made between type I blindsight, i.e., absolute blindness without conscious awareness, and type II blindsight, i.e., blindness with awareness but no visual qualia (Lau and Passingham, 2006). In fact, attempting to understand the intrinsic process governing this unconscious vision has been the foundation of many theories (Smythies, 1999; Kanemoto, 2004; Zeman, 2004).

### Fine Line between Type I and Type II Blindsight

It was in 1917 that the first evidence of a residual visual ability in the blindfield emerged when a patient reported visual sensation specifically to motion. This non absolute blindness sustained a form of visual qualia called the Riddoch Syndrome (Riddoch, 1917). Keeping this in mind, the notion of visual qualia is quite important to consider in blindsight studies, especially when considering blindsight type II because even if some kind of awareness remains it has to be dissociated from visual awareness (Ko and Lau, 2012) which we will discuss further on. Subsequently, the characterization and understanding of the dissociation between type I blindsight and type II is controversial, due to the fact that only a few studies have been able to demonstrate a correlation between the loss of the striate cortex, the takeover of secondary visual pathways and the state of visual consciousness (for review see Leopold, 2012). The problem that arises with respect to blindsight is to figure out which hypothesis could best explain the phenomenon: (1) a degraded normal vision; (2) an unconscious vision; and (3) a degraded abnormal vision.

1. Degraded normal vision occurs when visual stimuli are processed through the primary visual pathway, but do not reach the threshold of full visual awareness. In fact, in some cases, spared islands of the striate cortex explain the residual visual capabilities found in HH (Fendrich et al., 1992, 2001). However, several patients may present blindsight in the absence of a functional striate cortex (Morland et al., 2004; Ajina et al., 2015b; Mazzi et al., 2016), regardless of the state of awareness (Ffytche and Zeki, 2011). Though we should not overlook the importance of targeting vestiges of V1 in rehabilitation strategies, we must be able to stimulate the secondary visual pathways bypassing V1 potentially responsible for type I and II blindsight. Therefore, we need to understand the mechanisms governing the two forms of this phenomenon.
2. Unconscious vision has been showcased by proving that residual abilities in HH do not follow the same rules as it is qualitatively different from that of the conscious normal vision (Weiskrantz, 2009). In fact, for certain visual stimulations, the performance in the blind side is better than the one in the normal side (Trevethan et al., 2007). For example, unlike normal vision, performance in a task of exclusion is inversely correlated to the stimuli’s contrasts (Persaud and Cowey, 2008), there is a clear abnormal distinction between choice-forced and detection performances (Azzopardi and Cowey, 1997) and some physical attributes are processed in the blind hemifield, while others are not (Morland et al., 1999; Kentridge et al., 2007). Taken together, these studies provide robust evidences to the hypothesis that blindsight is different from normal vision and is not simply a form of degraded normal vision. However, they have assumed that this abnormal vision is unconscious, whereas an abnormal degraded vision could also explain the behavioral results. In fact, unconscious and degraded abnormal vision can both make reference to a vision qualitatively different from normal vision mediated by secondary neurophysiological correlates, but that differ in terms of conscious subjectivity and the nature of the sensation.
3. Some authors refute the theory of unconscious vision by stating that the residual visual capabilities are due to a degraded abnormal vision that does not reach the threshold of detection (Overgaard and Grünbaum, 2011; Mazzi et al., 2016). In GR and SL case studies, with complete lesion to the striate cortex, the perceptual awareness scale (PAS) was used to allow a subjective finer report based on four indices, instead of the usual scale of binary report (seen, not seen). This showed that patients tend to have a higher threshold to acknowledge that something is conscious if the criterions are not based on a scale of consciousness. In fact, after using the PAS, awareness was better than what the theory of unconsciousness would have predicted (Overgaard et al., 2008; Mazzi et al., 2016). They concluded that type I blindsight can be wrongly considered as unconscious; instead it seems that above chance level performance comes with conscious perception. Therefore GR and SL do not have blindsight, rather they have conscious vision. Their results agree with the continuum perception theory, where there is a correlation between increased visual sensitivity and higher brain activity. In the normal population, results are contradictory depending on the paradigm used. For example, when using masked stimuli, results tend to suggest that performance can’t exist without awareness and that if the reverse is often inferred, it is due to visual bias induced by inappropriate methodological tools measuring awareness. Moreover, even among the ideal model, performance is greater than awareness in a non-linear relationship where the threshold for perception is inferior to the one for awareness which could explain why in altered perception, the state of consciousness decreases more rapidly than the performance. Thus, even if performance is accompanied by awareness, the latter can be wrongly underestimated without the appropriate tools, since thresholds for explicit visual consciousness is not reached (Peters and Lau, 2015). Therefore, is it possible that blindsight consists of an abnormal conscious degraded vision mediated by secondary visual pathways? If this extrapolation is accurate, we nevertheless disagree with the conclusion of Mazzi et al. (2016) that SL’s residual abilities are due to conscious vison inducing visual qualia, thus inferring that in such case there is no such thing as blindsight. First, blindsight was employed to explain an ability which was phenomenologically different from blindness and sight, and can be referred to as the loss of visual function that is accompanied by altered “sight”. Second, SL had the feeling that something happened in her blindfield; however she couldn’t visually describe what she saw. Can we say that she showed a form of consciousness? Yes. Can we conclude that the nature of the feeling is visual? Not so much. The scale evaluated the following perceptual judgments as: “(1) no experience of the stimulus; (2) brief glimpse; (3) almost clear experience; and (4) clear experience” (Mazzi et al., 2016). There was never a reference to the nature of the visual stimuli, or even to what was seen, contrarily to the Riddoch phenomenon where visual qualia of motion was described (Riddoch, 1917). While we agree that the use of the PAS allows patients to “pay more attention”, subsequently giving more insight on residual abilities and potential tools to rehabilitation, it’s nevertheless insufficient to conclude on the nature of the awareness and the continuum scale of perception. In fact, others have also used a continuous scale to assess visual awareness and showed that either the visual stimuli presented in the attentional blink was completely perceived or not detected at all independently of stimuli visibility (Sergent and Dehaene, 2004a). The non-perceived stimuli in the attentional blink were correlated with suppression of the P300 wave, and a dynamic change in brain oscillations indicating that perception without consciousness has distributed neuronal correlates (Kranczioch et al., 2007). Unfortunately, the neuronal correlates underlying theses controversial residual abilities has yet to be explained.

### An Unsynchronized Framework for Blindsight

**Figure 1:**
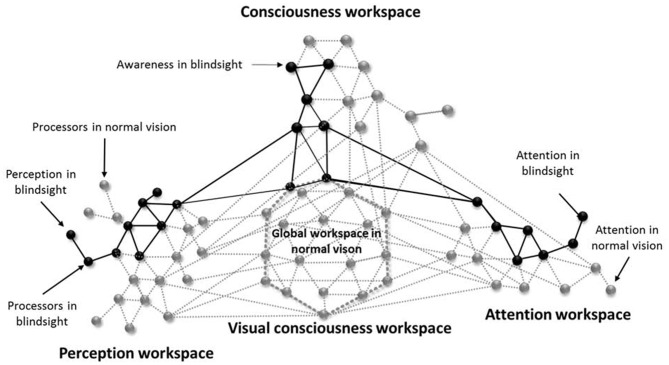
An illustrative schematic of the proposed model for unsynchronized framework for blindsight (inspired from Dehaene et al., 1998). The gray circles represent neuronal processors that are activated in normal vision and the gray lines their respective connections. The black circles illustrates the neuronal processors that underlies blindsight and the black lines their respective connections. Blindsight can be understood as an alteration in the perception and attentional systems, therefore inactivating the long-range workspace connectivity, global availability and conscious visual perception. The lack of visual awareness is due to a non-efficient global workspace. Awareness found in blindsight type II, could be linked to some long-range connectivity between the perception, attention and consciousness workspaces without activating the global workspace.

This article supports the global neuronal workspace framework as a model for conscious and unconscious vision (Sergent and Dehaene, 2004b). Thus, in alignment with promising views on “local” and “global” visual functions in blindsight (Silvanto, 2015), blindsight can be understood as a lack of synchronization in neuronal activity (Melloni et al., 2007) and rapid globalization for specific visual properties between altered perception processors (neuronal networks implicated in bottom-up activity and visual performances), attention processors (systems of complex neuronal association that allow perceptual information to access consciousness) and conscious processors (workspace neurons for awareness via top-down activity). Moreover, because the attention network can interact with the perception network without creating any kind of visual awareness, it is most probable that the perception workspace can send projections to the attention workspace without creating any attentional awareness. This phenomenon recently called attentional unawareness was hypothesized in blindsight patient for emotional stimuli, subsequent to studies in normal vision (for review see Diano et al., 2016). In this context, it would be conceivable to induce learning effect resulting in awareness, and moreover visual consciousness, if attentional and perceptual feedforward and feedback connections were simultaneously stimulated. This would lead to synchronization enhancement and would allow cascading amplification resulting in long-distance reciprocal connections and global availability (Dehaene et al., 1998). Indeed, attention is necessary to visual consciousness even if not sufficient (Kentridge et al., 2004; Schurger et al., 2008; Yoshida et al., 2012). Visual consciousness would be mediated by top-down activity through connections between higher and lower perception processors, as well as between perception, attention and conscious processors creating a global workspace. As a result, the inability of cortically blind people to describe what is presented in their blindfield could be linked to a lack of global availability of the global workspace due to inefficient looping among the altered perception and attention processors and interaction with the conscious network. Hence, according to the global workspace, blindsight could be mediated by secondary visual pathways that activate the neuronal perception and attention networks insufficiently and only locally, without sending long ranging connections to other networks in the brain therefore suppressing visual qualia which could explain the above-chance visual performances in choice-forced paradigms. Consequently, in type I blindsight, the neuronal network generates sufficient activity to process the stimulus, however the neuronal pattern required for phenomenal consciousness is insufficient. In type II blindsight, activity is sufficient to create a sense of awareness, perhaps due to the activation of local conscious processors, but it doesn’t reach the threshold for global availability (see Figure 1).

Nonetheless, all residual visual abilities found in CB are not necessarily due to blindsight, in the contrary it could be linked to degraded normal or abnormal vision, as we discussed previously. In reality multiple networks can co-exist, vary in function of the lesions and express themselves depending on the stimulation or given paradigm. In literature, the term blindsight lacks clarity because it refers to several types of visions, mechanisms and correlates all at once. Residual visual abilities are found only in a few individuals with CB. However, it is more than possible that co-existent residual secondary pathways arise together (Tamietto and Morrone, 2016), but when cortical alterations are too diffuse residual pathways aren’t activated strongly enough to induce residual vision. Therefore, a same individual could have multiple types of residual visual abilities or the potential to develop them with training. Thus, we propose the following terminology:

1. Degraded visual abilities consist of a degraded normal vision caused by vestiges of the striate cortex. It is linked to reduced performances and/or visual awareness that are qualitatively similar to normal vision but quantitatively poorer.
2. Blindsight consists of an unconscious vision mediated by secondary visual pathways bypassing V1 independent of visual awareness. It could be explained by inexistent (blindsight type I) or not optimal synchronization (blindsight type II) between the perception, attention and conscious networks.
3. Alternative visual abilities consist of an abnormal degraded vision which is qualitatively different from normal vision but is associated with visual awareness mediated by secondary visual pathways that can’t be explained by the activity of the striate cortex.

The idea is to understand how we can pass from blindsight type I, to blindsight type II to an alternative visual ability. In other terms how can we pass from a state of no awareness to a state of awareness and finally to visual awareness by stimulating secondary visual pathways?

## Neuronal Substrates Underlying The Framework of Blindsight

### Geniculo-Extrastriate Pathway: A Door to Perception

The geniculo-extrastriate pathway is a perfect candidate to our altered perception workspace. Its existence implies that V1 lesions do not lead to a complete degeneration of the LGN, and that koniocellular projections are sent to the secondary extrastriate regions, such as MT (Warner et al., 2010). In macaques with no striate cortex (eliminating the possibility of V1 islands) there is a causal link between the LGN and blindsight (Schmid et al., 2010). In fact, by presenting high contrasts stimuli in the blindfield, the authors have observed visual processing corresponding to blindsight, correlated with fMRI activations in several areas including extrastriate region MT. By inactivating the LGN, the neuronal activations and the residual detection skills were abolished (Schmid et al., 2010). Also in macaques, direct koniocellular projections were found between the LGN and MT corroborated by a retrograde technique of tracing and histological sections. In addition, a new neuronal population, not belonging to the koniocellular system, has been discovered in the intercalated layers of the LGN (Sincich et al., 2004). Interestingly, in humans MT (hMT+) acts similarly to V1 when presented with global motion (Ajina et al., 2015a). This highlights the role of existing subcortical visual pathways in blindsight, which was specifically and exclusively correlated with the presence of the geniculo-extrastriate pathway (Ajina et al., 2015b). However, in this study, blindsight was assessed with a motion task; it is possible that the correlation existed just for the geniculo hMT+ pathway because the psychophysical measure was specific to this pathway. Blindsight negative individuals were categorized as such using the same task, nonetheless they could have exhibited blindsight using saccadic localization of a brief visual flash, or using indirect methods where reaction time to stimuli presented in the normal field are enhanced by stimuli presented in the blindfield. We extrapolate a possible correlation between blindsight positive individuals derived from such paradigms and the colliculo-extrastriate pathway or interhemispheric connections between hMT+, respectively.

### Colliculo-Pulvinar-Extrastriate Pathway: A Door to Integration and Attention

The implication of the superior colliculus (SC) in blindsight is strongly corroborated with behavioral data. More specifically, the physical parameters of stimuli inducing blindsight correspond specifically to the inherent characteristics of the SC neurons (Leh et al., 2010; Tamietto et al., 2010). For example, the lack of projections from the short-wavelength sensitive cones of the retina towards SC neurons is associated with blindness to blue (Leh et al., 2010; Tamietto and de Gelder, 2010). Consequently, when the color blue is used instead of red or an achromatic visual stimulation, then blindsight and activations of the SC disappears. From a functional point of view, an association between the collicular pathway and type I blindsight was found in a hemispherectomized patient, as well as interhemispheric connections extending from the SC to the visual, parietal and prefrontal, areas (Leh et al., 2006). Even if the SC could relay to the extrastriate cortex via colliculo-geniculate projections (Harting et al., 1991), Lyon et al. (2010), have demonstrated projections from the SC to V3 and V5/MT throughout the pulvinar in macaques assessing the possibility that blindsight could be mediated by relays ranging from the SC to the pulvinar and the dorsal pathway similar to the magnocellular pathway involved in movement and ocular orientations. We postulate that the subcortical extrastriate pathway passing by the SC and the pulvinar serves attentional workspaces and can be enhanced with multisensory stimulations. In fact, bimodal neurons of the SC respond to audio-visual stimuli by fortifying extrastriate pathways (Stein and Rowland, 2011; Paraskevopoulos et al., 2012). Moreover, the SC is responsible for ocular movements in the centers of attention becoming faster and more accurate with repetitive multisensory stimulations (Corneil et al., 2002; Bell et al., 2005; Gingras et al., 2009). Training of the oculomotor track could allow a potential increase in allocation of attention in the blind hemifield, which is necessary to perception and visual consciousness. On another note, it seems that the pulvinar can perform higher order visual processing, as motion-selectivity and emotional processing for the former (Villeneuve et al., 2005; Maior et al., 2010) so can the SC, as Gestalt like analysis associated with faster responses for stimuli with specific configuration and numerosity (Celeghin et al., 2015a; Georgy et al., 2016). Other studies demonstrated activations, projections and connections from the SC and the pulvinar to the amygdala when unconscious visual emotional stimuli occurred in hemispherectomized patients (de Gelder et al., 1999; Morris et al., 2001; Tamietto et al., 2012; Celeghin et al., 2015b). Therefore specific perceptual training could directly target these structures and reinforce subcortical pathways bypassing V1, hence the idea of combining different types of visual training.

### Multiple Workspaces of Consciousness

We hypothesize that consciousness and moreover visual consciousness is mediated by multiple workspaces interacting together. Conscious processors can be mediated by interactions of the fronto-parietal and prefrontal network (Zeman, 2004; Persaud et al., 2011) with higher visual areas (Dehaene and Changeux, 2011), and visual conscious processors by the thalamic reticular network (Min, 2010). Consciousness, and more specifically visual consciousness can be achieved with feedforward and feedback connections from higher to lower visual areas (for review see Urbanski et al., 2014). An alteration in feedback loops and synchronization between high cognitive areas and visual areas could lead to a lack of awareness (blindsight type I). Between higher and lower visual areas inefficient long ranging connections could lead to the lack of visual awareness found in type II blindsight. This blindsight model is subsequently the result of altered local workspaces that take over when a normal global network degenerates (Silvanto, 2015). Therefore, connectivity between new workspaces of perception and abnormal workspace of consciousness are weak and non-specific, due to a lack of visual learning reflected by a lack of appropriate synchronization. This unsynchronized framework between posterior and more anterior areas diminishes the visual sensitivity for motion stimuli in healthy subjects demonstrating precisely the effects of synchronization on V5 (Romei et al., 2016). Hence, the idea is to employ neurorehabilitation to target residual pathways passing by V5/MT, induce new connectivity between interhemispheric V5/MT areas (Bridge et al., 2008; Silvanto et al., 2009) and functional interactions within the lesioned hemisphere (Huxlin, 2008).

## A Model of Combined Interventions Reinforcing The Global Framework of Blindsight

### Importance of the Subacute Period

This section reports the estimated tools to promote plasticity following a CB and increase potential recovery of functional vision. First, future researches should emphasize the importance of stimulating visual pathways in the subacute period following the lesion (Alber et al., 2017) to notably reduce the degenerations of subcortical tracks (Nijboer et al., 2013) and increase the chances of visual improvement (Keller and Lefin-Rank, 2010). In fact, spontaneous restoration in the subacute phase is associated with a reactivation of V1, a restoration of the ipsilateral optical radiations and a progressive recovery of visual functions (for review see Matteo et al., 2016). An increase in spontaneous restoration could be induced with reinforcement of the residual tracks and recruitment of new ones by activating interhemispheric connections.

### The Value of Interhemispheric Connections

It has been demonstrated that subsequent to a striate lesion, reorganization of the cerebral cortex in favor of the intact hemisphere induces V5/MT of the ipsilateral hemifield to project to V5/MT of the contralateral hemifield after stimulation of the blind field (Bridge et al., 2008). Moreover, in the lesioned hemisphere, there are areas that respond to stimulations presented in the normal hemifield, but not to stimulations in the blind hemifield (Kavcic et al., 2015). Simultaneous stimulation of the two hemifields could produce an effect of learning, allow for ipsilesional reorganization (Celeghin et al., 2015a) and would be essential to regain visual perception (Silvanto et al., 2007). Contralesional V5/MT activation induced by repetitive stimulation of the normal hemifield would allow reorganization and potentiation of the ipsilesional V5/MT via interhemispheric connections, and therefore gain new normal functionality instead of the “V1 like functions”. In fact, we postulate that connections from the contralesional to the ipsilesional V5/MT could lower the threshold to induce global availability within the lesioned hemisphere by activating new processors in the lesioned hemisphere and/or by interhemispheric synchronization between workspaces. These results added to the proposed neuronal substrates of the global workspace seem to lead to the notion that V5/MT could be considered as the crossroad of residual abilities and should therefore be the central key to rehabilitation. Multisensory bottom-up activations mediated by the colliculo-extrastriate pathway could lead to V5/MT enhancement without the need of attentional processes.

### Enhancing Attention with Audio-Visual Training

Reorganization following audio-visual stimulations allows a decrease of the ipsilesional attentional bias showed by a reduction in P300 amplitude (Dundon et al., 2015b), potentially moving the attentional capacities towards the blind hemifield. The role of the SC in this attentional process is particularly important, since such an effect is obtained by the intermediary of saccadic movements. This enhancement of attentional capacities, without prior visual attention needed, is exactly why rehabilitation tools should include audio-visual stimulation training (for review see Grasso et al., 2016) which has been associated with an improvement in visual detection and exploration (Bolognini et al., 2005; Leo et al., 2008; Passamonti et al., 2009), and in life quality (Roth et al., 2009). Although compensation therapies proved their reliability over more than two decades (Kerkhoff et al., 1992), they are still underestimated, due to very little clinical evidence of their impact (Pollock et al., 2011). For this reason, the use of multisensory bottom-up training in association with top-down training could lead to a higher chance of improving visual detection, localization and recognition.

### Re-Establishing Perception with Restitution Training

Restitution techniques are effective if they aim typical visual attributes training specific to blindsight to expand over a large spectrum of visual characteristics and functions. For example, a transfer of information can be achieved by presenting simultaneous and diversified stimulations in the blindfield accompanied by temporal and spatial cues (Kentridge et al., 1999). As well, it would be possible to improve conscious visual detection performances with training of residual visual abilities (Chokron et al., 2008), to improve visual functions that are initially outside of the spatiotemporal band of blindsight by using double stimulations including complex motion and static stimuli presented in different positions in the blind field (Das et al., 2014). This improvement in perception is obtained when a transfer of information processing happens between different stimuli and experimental conditions (Huxlin et al., 2009), implying that the perception workspace is capable of great plasticity when it is targeted via different mechanisms. Restitution tools must therefore target multiple functions used in perception, that is to say: detection, localization, identification and discrimination, as well as functions used in consciousness, that is to say: a judgment on the nature and the level of visual consciousness (Sahraie et al., 2013). Moreover, it is possible with repeated stimulation to increase visual sensitivity (Sahraie et al., 2006, 2010; Trevethan et al., 2012). This change in subjective awareness linked to the performance, highlights the possibility of a transfer from an unconscious vision (type I blindsight), to a state of awareness (type II blindsight), hence to a potential visual qualia (vision), which is encouraging in regards of rehabilitation tools (Sahraie et al., 2013). Taken together, these results imply that to gain vision, we have to trigger long-term plasticity by targeting multiple pathways and mechanisms together creating a synchronous activity through multiple processors of the blindsight framework. Thus, we endorse a combined strategy using multisensory compensation and restitution.

### Potential Effects of a Combined-Training within a Global Subcortical Framework of Blindsight

**Figure 2:**
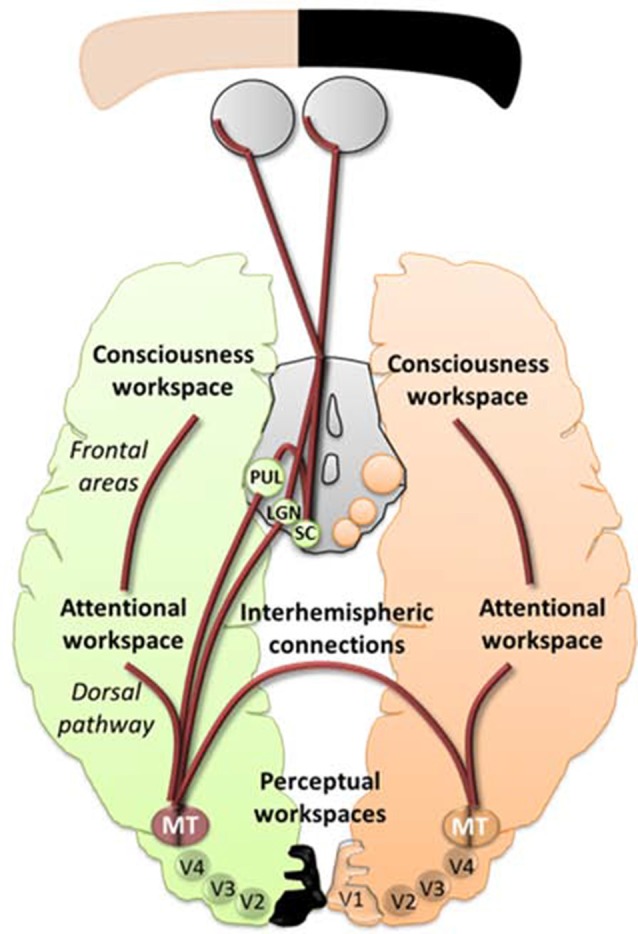
An illustrative schematic of the proposed hypothesis of the pathways involved in blindsight within the model of global workspace. In peach and green are represented the normal and lesioned hemispheres and subcortical areas projecting towards their respective hemispheres. The brown lines represent feedforward and feedback projections between workspaces. Enhancing the projections from the superior colliculus (SC)/pulvinar and the Lateral Geniculate Nucleus (LGN) to V5/MT and interhemispheric connections between V5/MT could allow synchronization between different areas, including the extrastriate regions, the dorsal pathways and the frontal areas, thus leading to more efficient interactions between lower and higher visual areas resulting in long-distance reciprocal connections and cascading amplification in the conscious workspace.

Audio-Visual Scanning Training could allow feedforward interactions between the SC and V5/MT (Dundon et al., 2015a), causing V5/MT to increase its functional activity and potential to make stronger connections in the attentional workspace. When applied with restitution training, the increase in functionality could optimally reinforce the tracks between the LGN and V5/MT in the perception workspace (Ajina et al., 2015b), leading to more efficient interactions with lower and higher visual areas resulting in long-distance reciprocal connections and cascading amplification in the conscious workspace (see Figure 2). Therefore, a stimulation of the altered blindsight framework would allow attention and perception to enhance each other leading to a better access to consciousness by a decrease in the threshold of visual attention and discrimination. Lowering these thresholds implicates that the visual properties of a stimulus are prompt to be accessible to different areas of the brain making them more easily perceived and processed permitting awareness. This will be reflected by higher synchronization of neural activity in visual and higher cognitive areas which will induce global availability and possibly lead to conscious visual perception (Melloni et al., 2007). Finally, although the main focus of this review covered visual training, we can’t omit the potential benefit of pharmacological interventions (Gratton et al., 2017) and novel tools for neuromodulation used alone (Gall et al., 2015) or combined with vision restauration strategies, e.g., VRT with dtCS (Plow et al., 2011; Alber et al., 2017), that could target in different ways the global workspace. However, let’s keep in mind that prior to using any kind of neurostimulation it would be essential to use an efficient visual training that could facilitate rehabilitation at home.

## Conclusion

The problem is to know how visual therapies can target residual visual abilities when neurophysiological correlates are so divergent between patients. Can we really use what we know of blindsight to develop rehabilitation tools? Our review explains how combined rehabilitation tools using visual training can enhance blindsight by targeting an inefficient global framework. Blindsight, defined as an unconscious residual visual ability, can come with or without awareness, but except in rare cases, doesn’t elicit visual awareness (Balsdon and Azzopardi, 2014). The reason why some patients may not present residual vision or awareness could include an inability to allocate sufficient attention to the information presented in the blind hemifield and to access their own state of consciousness. By understanding blindsight within the global workspace theory (Sergent and Dehaene, 2004b), we can define the lack of visual awareness as a lack of neuronal synchrony and global availability between inefficient workspaces of attention, perception and consciousness that we can target and optimize with rehabilitation tools. Therefore, it would be possible to pass from a state of no awareness (type I blindsight) to a state of awareness (type II blindsight) to a state of visual awareness (alternative visual abilities) by moving the thresholds of attention, perception and consciousness via stimulation of the colliculo and geniculo-extrastriate pathways and creating connections between different processors. By doing so, we could target higher visual areas as V5/MT, induce loops with higher cognitive areas, synchronization of neuronal activity and global availability, and potentially it would lead to visual consciousness. These mechanisms can be targeted optimally in the subacute phase, using interhemispheric stimulations, Audio-Visual Scanning Training and combined restitution strategies, where several processes are enhanced at the same time inducing learning transfer and promoting the brain reorganization. The establishment of new guidelines in rehabilitation tools targeting the global framework of blindsight can lead to clinical intervention tools applicable to the majority of CB patients.

## Author Contributions

FL and VH: equal contribution for the literature search and article preparation.

## Conflict of Interest Statement

The authors declare that the research was conducted in the absence of any commercial or financial relationships that could be construed as a potential conflict of interest.
